# Nationwide hospitalizations of patients with down syndrome and congenital heart disease over a 15-year period

**DOI:** 10.1007/s00431-024-05542-2

**Published:** 2024-04-16

**Authors:** Alvise Guariento, Claudia Cattapan, Giulia Lorenzoni, Giulia Guerra, Ilias P. Doulamis, Giovanni di Salvo, Dario Gregori, Vladimiro L. Vida

**Affiliations:** 1 Division of Pediatric and Congenital Cardiac Surgery, Department of Cardiac, Thoracic and Vascular Sciences and Public Health, University of Padua, Padua, Via Giustiniani 2, 35100 Italy; 2 Divsion of Biostatistics, Epidemiology and Public Health, Department of Cardiac, Thoracic and Vascular Sciences and Public Health, University of Padua, Padua, Italy; 3 Department of Surgery, Johns Hopkins University School of Medicine, Baltimore, MD USA; 4 Division of Pediatric Cardiology, Departments of Women’s and Children’s Health, University of Padua, Padua, Italy

**Keywords:** Down syndrome, Congenital heart disease, Nationwide hospitalizations, Outcomes

## Abstract

Down syndrome is one of the most common genetic diseases, generally associated with an increased probability of congenital heart diseases. This increased risk contributes to escalated levels of morbidity and mortality. In this study, we sought to analyze nationwide data of pediatric and adult patients with Down syndrome and congenital heart disease over a 15-year period. Data obtained from the hospital discharge form between 2001 and 2016 of patients diagnosed with Down syndrome in Italy and at least one congenital heart disease were included. Information on 12362 admissions of 6527 patients were included. Age at first admission was 6.2 ± 12.8 years and was a predictor of mortality (HR = 1.51, 95% CI 1.13–2.03, *p* = 0.006). 3923 (60.1%) patients underwent only one admission, while 2604 (39.9%) underwent multiple (> 1) admissions. There were 5846 (47.3%) admissions for cardiac related symptoms. Multiple admissions (SHR: 3.13; 95% CI: 2.99, 3.27; *P* < 0.01) and cardiac admissions (SHR: 2.00; 95% CI: 1.92, 2.09; *P* < 0.01) were associated with an increased risk of additional potential readmissions. There was an increased risk of mortality for patients who had cardiac admissions (HR = 1.45, 95% CI: 1.08–1.94, *p* = 0.012), and for those who underwent at least 1 cardiac surgical procedure (HR = 1.51, 95% CI 1.13–2.03, *p* = 0.006).

*Conclusions*: A younger age at first admission is a predictor for mortality in patients with Down syndrome and congenital heart disease. If patients undergo more than one admission, the risk of further readmissions increases. There is a pivotal role for heart disease in influencing the hospitalization rate and subsequent mortality.

**Table:** 

**What is Known:** • *Down syndrome individuals often face an increased risk of congenital heart diseases.* • *Congenital heart diseases contribute significantly to morbidity and mortality in Down syndrome patients.*	
**What is New:** • *This study analyzes nationwide data covering a 15-year period of pediatric and adult patients in Italy with Down syndrome and congenital heart disease.* • *It identifies a younger age at first admission as a predictor for mortality in these patients, emphasizing the criticality of early intervention.* • *Demonstrates a correlation between multiple admissions, particularly those related to cardiac issues, and an increased risk of further readmissions, providing insights into the ongoing healthcare needs of these individuals.*

**What is Known:**

• *Down syndrome individuals often face an increased risk of congenital heart diseases.*

• *Congenital heart diseases contribute significantly to morbidity and mortality in Down syndrome patients.*

**What is New:**

• *This study analyzes nationwide data covering a 15-year period of pediatric and adult patients in Italy with Down syndrome and congenital heart disease.*

• *It identifies a younger age at first admission as a predictor for mortality in these patients, emphasizing the criticality of early intervention.*

• *Demonstrates a correlation between multiple admissions, particularly those related to cardiac issues, and an increased risk of further readmissions, providing insights into the ongoing healthcare needs of these individuals.*

## Introduction

Down syndrome (also referred to as Trisomy 21) stands as the most prevalent genetic disorder compatible with life, boasting an estimated prevalence of 5 cases per 10,000 individuals in Europe [1–4]. Individuals with Down syndrome face an elevated susceptibility to associated diseases, which contribute to increased hospital admissions and reduced life expectancy. Notably, the connection between congenital heart disease (CHD) and Down syndrome patients (DSP) is significantly stronger compared to the general population [5–7]. In DSP, the prevalence of this association is notably higher (50% in DSP versus 1% in the general population), and certain types of malformations, such as intracardiac septal defects, are more frequently observed [8–10].

CHDs significantly elevate both mortality and morbidity among DSP, emerging as the foremost contributor to postnatal mortality within this population. Furthermore, the admission rate is notably elevated in DSP individuals even without CHD [8]. Numerous factors contribute to the heightened risk in the context of CHD. Indeed, DSP often possess compromised immune systems, rendering them more vulnerable to respiratory infections such as pneumonia and bronchitis. Down syndrome is linked to differences in the immune system, blood-related conditions like leukemia, and endocrine disorders that can affect overall well-being and lifespan. It is crucial to highlight, however, that medical and surgical approaches to managing CHDs has resulted in extended life expectancy and a substantial reduction in mortality rates associated with pediatric cardiac surgery [7, 11]. Nevertheless, the economic and emotional strain on families remains a considerable challenge for this population.

Examining both pediatric and adult patients affected by Down syndrome and CHD offers a valuable opportunity to formulate comprehensive management guidelines aimed at enhancing the overall quality of life within this group and potentially alleviating societal burdens. This study endeavors to analyze nationwide hospital data involving individuals with Down syndrome and CHD over a 15-year period in Italy.

## Materials and methods

### Study design

A retrospective, single-cohort, observational study was undertaken encompassing all pediatric and adult patients diagnosed with both Down syndrome and CHD who had undergone at least one hospital admission in Italy. The study period extended from January 1, 2001, to December 31, 2016.

The data utilized in this study were obtained from the Hospital Discharge Form (HDF), maintained by the Italian Ministry of Health, and released for research purposes after a waiting period of 5 years. The HDF contains a comprehensive range of personal, clinical, and admission-related information for each patient, with a unique identification code assigned to every individual. Diagnoses and procedures recorded in the HDF were systematically classified based on the International Classification of Diseases − 9th Revision - Clinical Modification System (ICD-9-CM).

### Data classification

Utilizing the ICD-9-CM code, hospital admissions were categorized into two main groups: cardiac and non-cardiac. These groups were subsequently subdivided into surgical and non-surgical. Patients were further stratified based on the presence of one or more CHDs. Within this subgroup, cardiac diagnoses were categorized based on the clinical significance of the specific condition. Non-cardiac diagnoses were systematically classified by considering the presence or absence of associated comorbidities. This categorization involved a thorough assessment of additional health conditions that might coexist with the primary cardiac diagnosis. Discharge outcomes were differentiated as either patient release or mortality. Lastly, a start and end variable were established, signifying the initiation and culmination of an event-free time period.

From the overall population, we derived three distinct datasets:

1. Total population: This dataset encompassed all patients hospitalized during the period from 2001 to 2016. It included assessments of both cardiac and non-cardiac diseases, recording the number and nature of cardiac and non-cardiac conditions, as well as the duration and type of hospital stays.
2. Admissions Related to CHD Requiring a Procedure (surgical or cath-based): In this dataset, we specifically focused on hospital admissions related to CHDs that necessitated procedures. Our evaluation encompassed the number and types of cardiac diagnoses, the frequency of hospital admissions, and the ultimate outcomes of these admissions (discharge or death).
3. DSP with CHD born between 2001 and 2016: This dataset focused on individuals with Down syndrome and a CHD diagnosis, born within the timeframe of 2001 to 2016. Here, we detailed start and end variables, and we assessed length and type of hospital stay, diagnosis, and the outcome of the admission (discharge or death).

### Statistical analysis

Continuous variables are expressed as mean and standard deviation, while categorical data are presented using frequencies and percentages. Normality of all continuous variables was tested with the parametric test Shapiro-Wilk and assessed with graphical methods.

To evaluate the trends in different diseases and their respective mortality rates, we conducted a comparison between the first eight years of the study period and the following years.Overall survival was analyzed using Kaplan-Meier curves with 95% confidence intervals (CI) estimated using Greenwood’s formula. Kaplan-Meier curves are presented with the number of patients at risk during follow-up. Univariate Cox proportional hazards regression model analyzes were used to analyze mortality risk factors adjusting for baseline covariates with results presented as hazard ratios (HRs) with 95% CI and P-values. Time zero for mortality analysis was defined as the date of birth.

The analysis of risk factors related to admissions was conducted using an innovative statistical methodology. This approach integrates time-to-event analysis, accounting for repeated events within the same patient. Mortality was considered as a concurrent outcome, employing informational censorship. Specifically, a modulated renewal analysis with regression of the competing risks was applied to provide a comprehensive understanding of the intricate relationships between various factors and their impact on admissions and mortality within the studied population [12].

Results from univariate competing risk regression models are presented using the HR sub-distribution (crude and adjusted, respectively) with corresponding 95% CI and P-values. Time to event was used as a predictor variable for patients who had > 1 admission to evaluate the association between the time since the previous hospital admission and the risk of each outcome.

Statistical analysis was performed using Stata software version 18.0 (Stata Corp LLC, College Station, Texas).

## Results

### Total population data

Data was gathered concerning a total of 13,269 admissions that occurred between 2001 and 2016 for patients in Italy with both Down syndrome and CHDs. However, 907 of these admissions were omitted from the analysis due to the identification of anomalous and contradictory data. These discrepancies were likely attributed to errors during the compilation of the HDF.

The analysis covered a total of 12,362 admissions, involving 6,527 individuals with Down syndrome and congenital heart disease (CHD). Within this cohort, 3,208 were females (49.1%), and 3,319 were males (50.9%). Most of the patients (5,662 individuals, accounting for 86.7%) were born in Italy, while the remaining individuals immigrated to Italy after birth. The average age at the time of the initial admission was 6.2 ± 12.8 years. Notably, 7256 (58.7%) of the hospital admissions occurred within the first year of life. Pediatric admissions comprised 10,701 cases (86.6%), with 1661 admissions (13.4%) occurring during adulthood. The mean duration of hospital stay was 11.6 ± 20.2 days, exhibiting a higher value for patients under the age of 18 (12.3 ± 19.6 days compared to 7.0 ± 12.5 days for adults, *p* < 0.001).

**Fig. 1:**
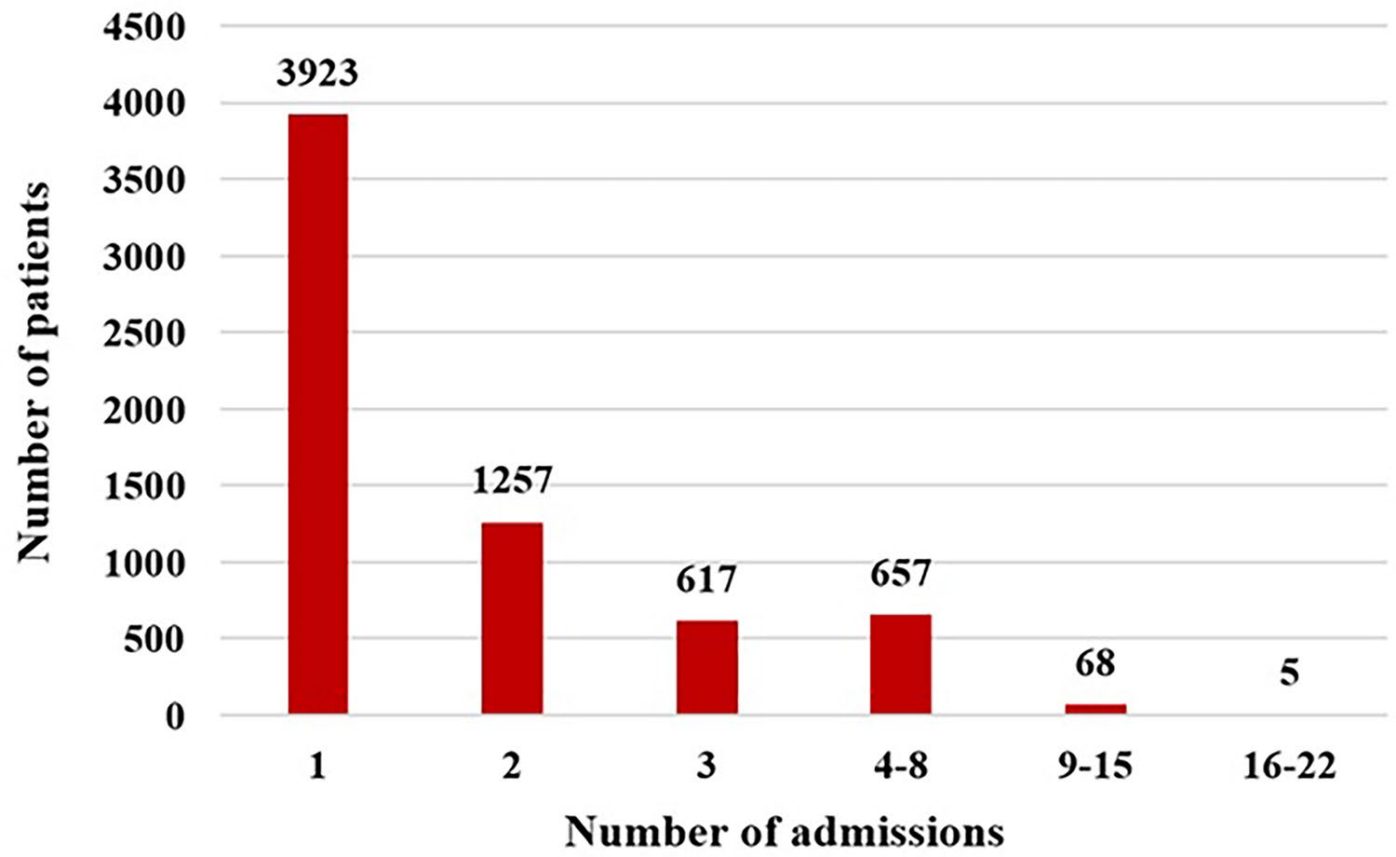
Number of admissions per patient over a 15 year period

The predominant cardiac diagnosis among patients was atrioventricular septal defect (AVSD), which accounted for 65.1% of cases, totaling 4250 patients. Following closely, the second most prevalent diagnosis was ventricular septal defect (VSD), with 3247 patients, constituting 49.7% of the total. Out of the total, 3923 patients (60.1%) experienced a single hospital admission, whereas 2604 patients (39.9%) underwent multiple (> 1) admissions (Fig. 1). Cardiac-related admissions accounted for 5846 cases (47.3%). Within this subset, 1687 admissions (13.6%) necessitated surgical procedures, while 4149 (33.6%) were cardiac medical admissions. Non-cardiac admissions totaled 6516 (52.7%), with non-cardiac medical admissions predominating (48.5% of medical non-cardiac admission in contrast to 4.21% surgical non-cardiac adimission). An overall occurrence of in-hospital mortality was reported in 283 patients (2.3%).

### Admissions related to congenital heart disease requiring a procedure

In total, there were 2005 hospital admissions directly linked to CHDs that necessitated a procedure. Among these, a substantial majority (1687 hospitalizations, 84.1%) were correlated with surgical procedures (Tables 1 and 2). AVSD repairs stood out as the most frequently performed surgical intervention (constituting 49.7% of cases), followed by VSD closures (26.4%), atrial septal defect (ASD) closures (4.2%), and mitral valve surgeries (4.2%). Notably, most of these cardiac surgical procedures were conducted on pediatric patients, accounting for 1911 cases (95.3%). Conversely, 318 admissions (15.8%) centered around percutaneous procedures. Within this context, the most prevalent procedure was the closure of a patent ductus arteriosus (PDA) (representing 64.2% of cases), followed by ASD closures (13.5%), and aortic or pulmonary valvuloplasty (12.9%).

**Table 1:** Total Population Data

	**Mean (Standard Deviation) Or N (%)**
Total Patients	6527 (100.0%)
Male	3319 (50.9%)
Female	3208 (49.1%)
Total Admissions	12,362 (100.0%)
Pediatric (< 18 Years)	10,701 (86.6%)
Adult (> 18 Years)	1661 (13.4%)
Cardiac Admissions	5846 (47.3%)
Cardiac Surgical Admissions	1687 (13.6%)
Cardiac Medical Admissions	4149 (33.6%)
Non-Cardiac Admissions	6516 (52.7%)
Non-Cardiac Surgical Admissions	5996 (48.5%)
Non-Cardiac Medical Admissions	519 (4.2%)
Patients With Single Admission	3923 (60.1%)
Patients With Multiple Admissions	2604 (39.9%)
Age At First Admission (Years)	6.2 (12.8)
Length Of Stay (Days)	11.6 (20.2)
Pediatric (< 18 Years)	12.3 (19.6)
Adult (> 18 Years)	7.0 (12.5)
In-Hospital Mortality (Number of Patients)	283 (2.3%)

**Table 2:** Type Of Procedures Related to Congenital Heart Diseases

	**SURGICAL**		**PERCUTANEOUS**		**TOTAL**	
	**N**	**%**	**N**	**%**	**N**	**%**
AVSD repair	838	49.7	0	0.0	838	41.8
VSD closure	447	26.5	14	4.4	461	23.0
PDA closure	20	1.2	204	64.2	224	11.2
ASD closure	71	4.2	43	13.5	114	5.7
TOF Correction	71	4.2	0	0.0	71	3.5
Mitral Valve Surgery	70	4.2	0	0.0	70	3.5
Aortic or Pulmonary Valvuloplasty	0	0.0	41	12.9	41	2.0
Systemic-To- Pulmonary Artery Shunt	29	1.7	0	0.0	29	1.5
Double Chamber Right Ventricle	26	1.5	0	0.0	26	1.3
Others	115	6.8	16	5.3	131	6.5
**Total**	**1687**	**100.0**	**318**	**100.0**	**2005**	**100.0**

*ASD* atrial septal defect, *AVSD* atrio-ventricular septal defect, *PDA* patent ductus arteriosus, *TOF* tetralogy of Fallot, *VSD* ventricular septal defect

### Population with a congenital heart disease born between 2001 and 2016

A sub-analysis was conducted on 4872 individuals with Down syndrome and CHD, born between 2001 and 2016. This subset contributed a total of 9142 hospital admissions to the study. Out of these admissions, 4087 (44.7%) were attributed to cardiac procedures. Among the patients, 3607 (74.0%) had a single CHD diagnosis. AVSD emerged as the primary diagnosis, accounting for 1252 (25.7%) of cases, with a higher prevalence among females (55.4%) (Table 3). Conversely, a male preponderance was noted among tetralogy of Fallot (TOF) patients (54.1%). The second most common diagnosis was VSD, accounting for the 1247 (25.6%) of patients.

**Table 3:** Primary congenital heart disease of patients born over a 15-year period (2001–2016)

	**NUMBER**	**PERCENTAGE**
AVSD	1331	27.3%
VSD	1254	25.7%
ASD	1050	21.6%
PDA	689	14.1%
TOF	146	3.0%
Non-Specified Anomaly	101	2.1%
Aortic Coarctation	28	0.57%
Other	273	5.6%
**Total**	**4872**	**100.0%**

*ASD* atrial septal defect, *AVSD* atrio-ventricular septal defect, *PDA* patent ductus arteriosus, *TOF* tetralogy of Fallot, *VSD* ventricular septal defect

**Fig. 2:**
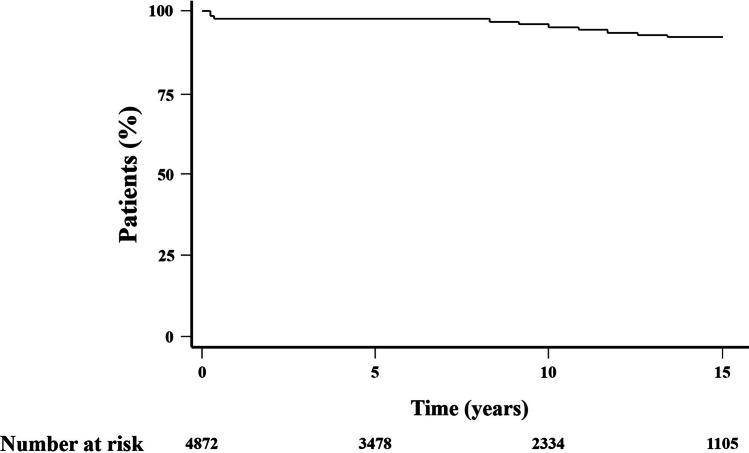
Kaplan-Meier Survival of a Nationwide Population of Patients with Down Syndrome and Congenital Heart Disease Born Between 2001 and 2016

A general decline in the number of patients diagnosed with both Down syndrome and CHD was noted between the first and period of the study. Nevertheless, in the comparison between the initial eight years of the study period and the subsequent years for the two primary diagnoses, no statistically significant differences were observed (*p* = 0.4).Initial admissions were non-cardiac related for 3122 patients (64.1%). However, when a cardiac admission was necessitated, 450 patients (9.2%) underwent a cardiac surgical procedure. The study documented in-hospital deaths among 189 patients (3.9%), with 171 (90.0%) of these deaths occurring in patients less than 1 year old (Fig. 2).

A single admission was observed in 2896 (59.4%) patients, while 946 (19.4%) and 496 (10.2%) underwent two and three admissions, respectively. The remaining 534 patients (11.0%) underwent four or more admissions. Most of the initial admissions (4391 patients, 90.1%) occurred within the first year of life.

**Fig. 3:**
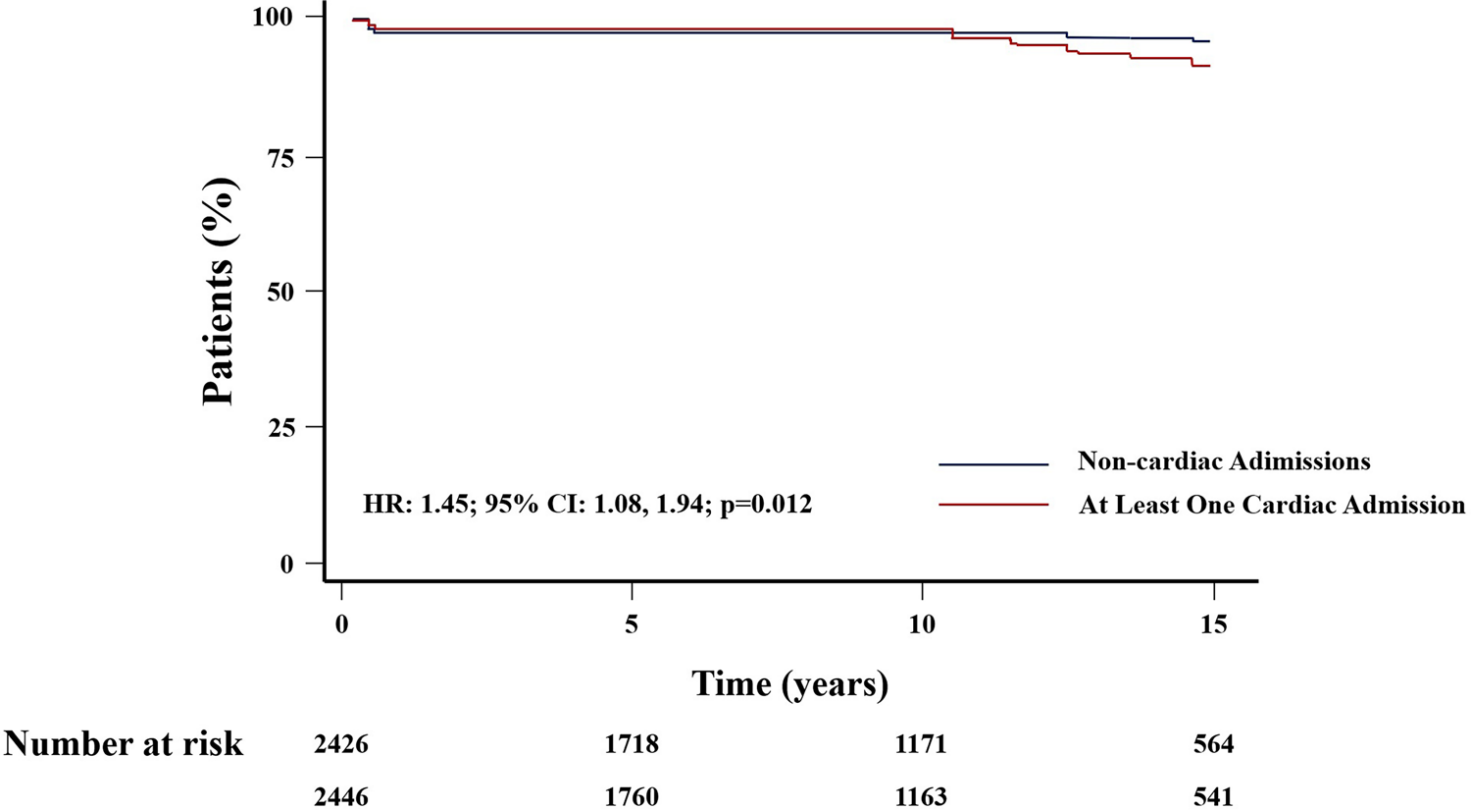
Kaplan-Meier Survival Based on The Type of Admission

**Fig. 4:**
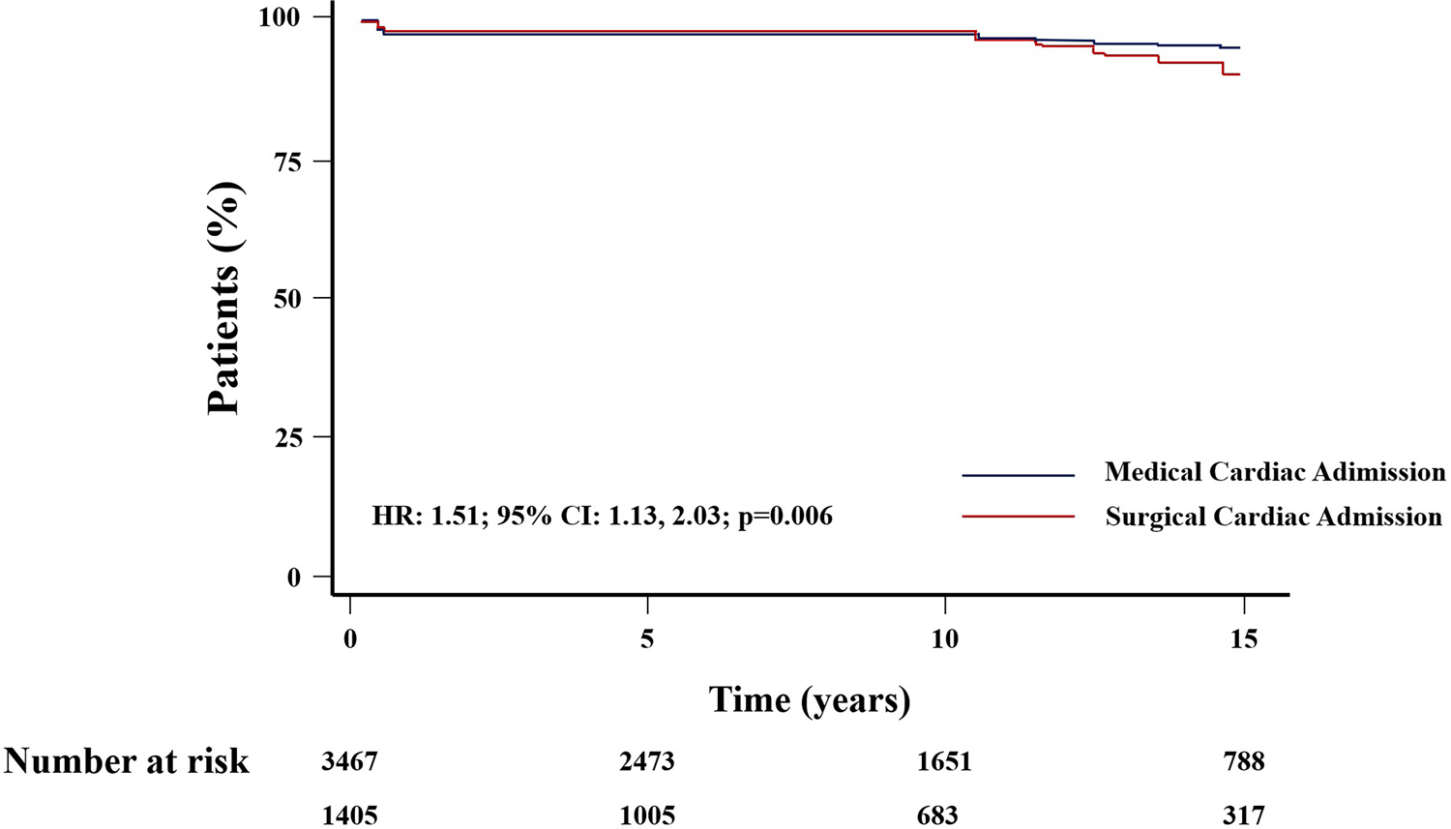
Kaplan-Meier Survival Based on The Type of Cardiac Admission

**Fig. 5:**
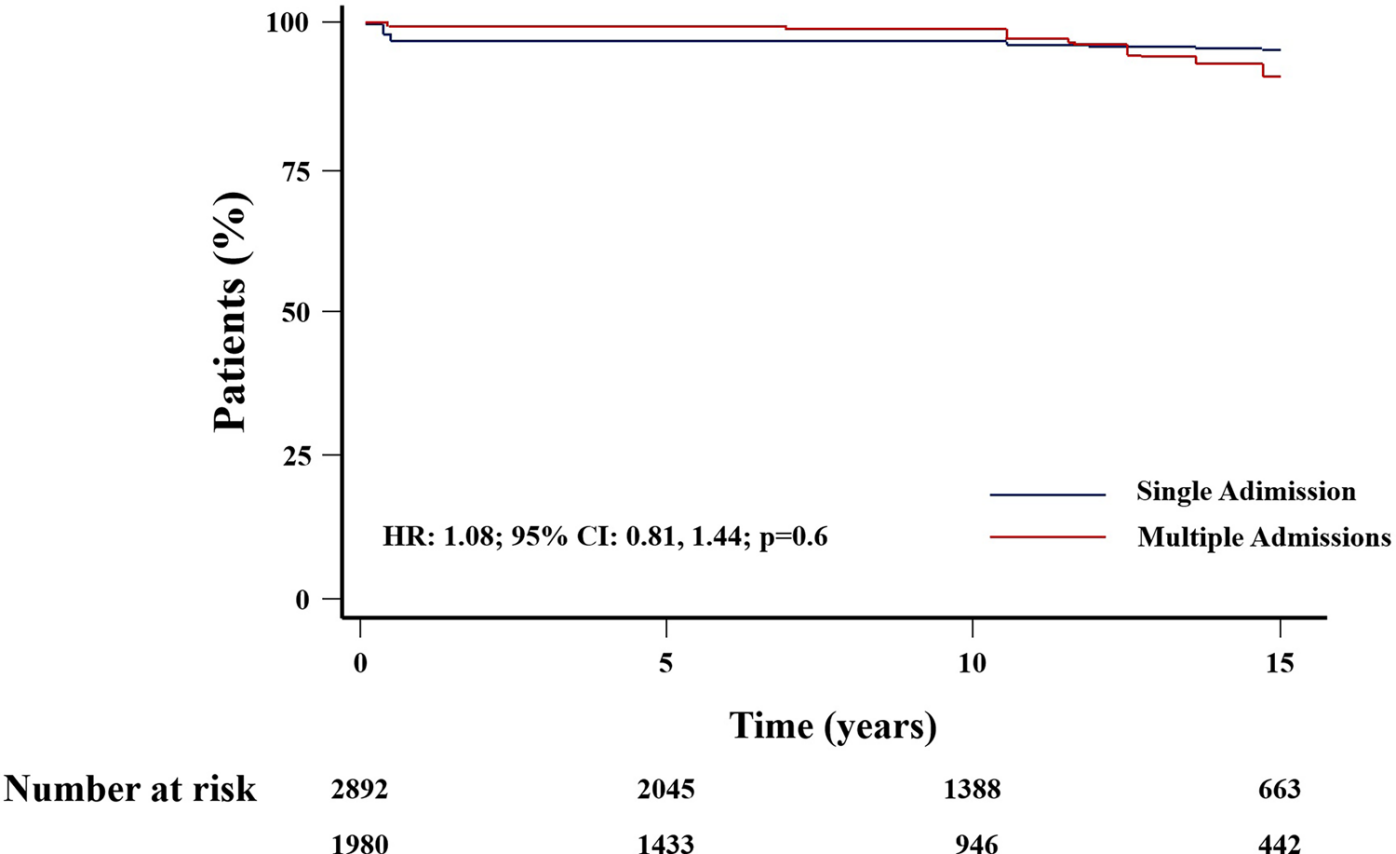
Kaplan-Meier Survival Based on The Number of Admissions

**Fig. 6:**
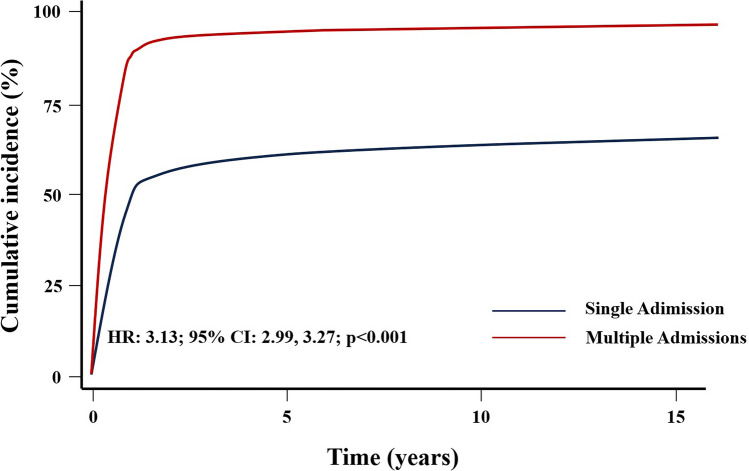
Competing Risk Modulated Renewal Regression Based on The Number of Admissions

**Fig. 7:**
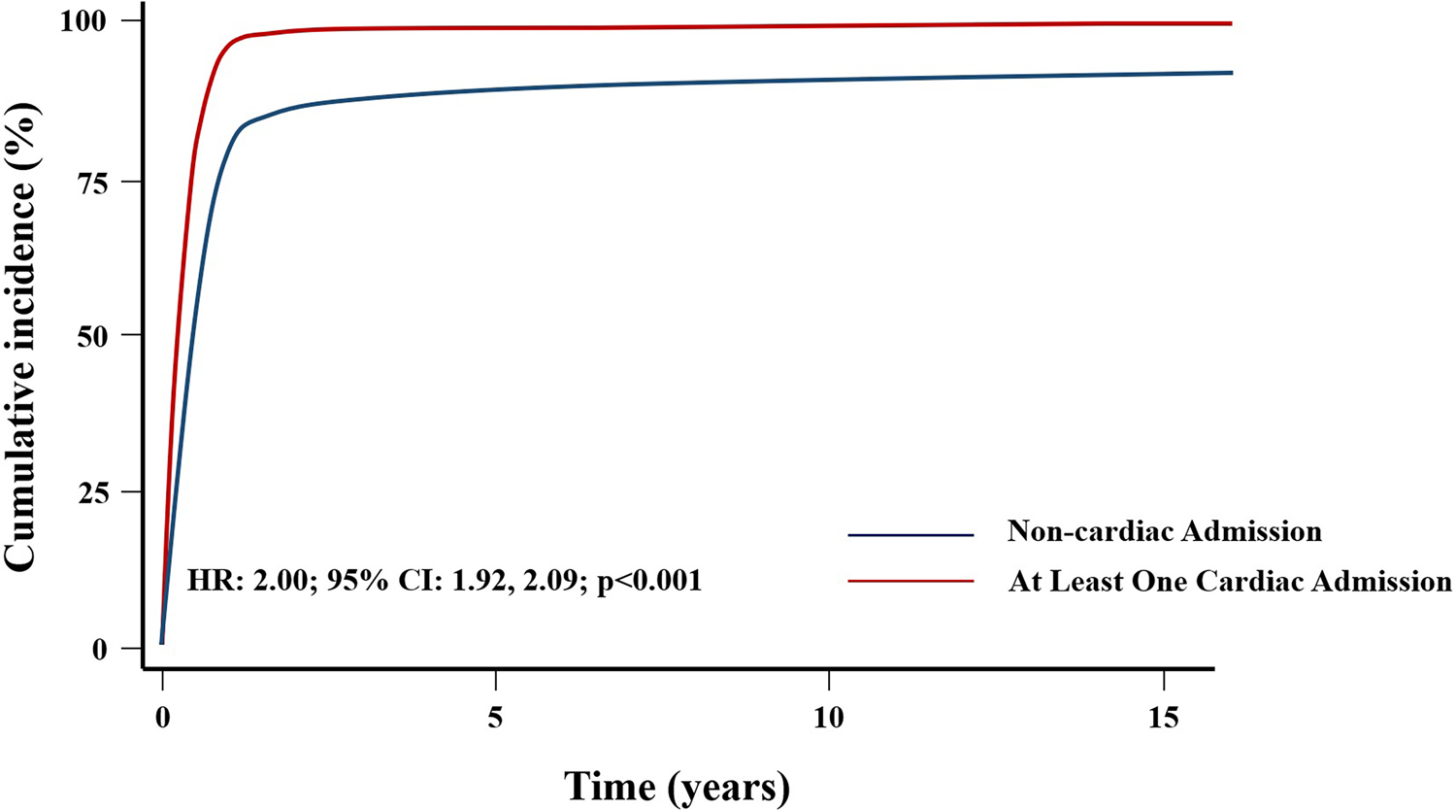
Competing Risk Modulated Renewal Regression Based on The Type of Admission

**Fig. 8:**
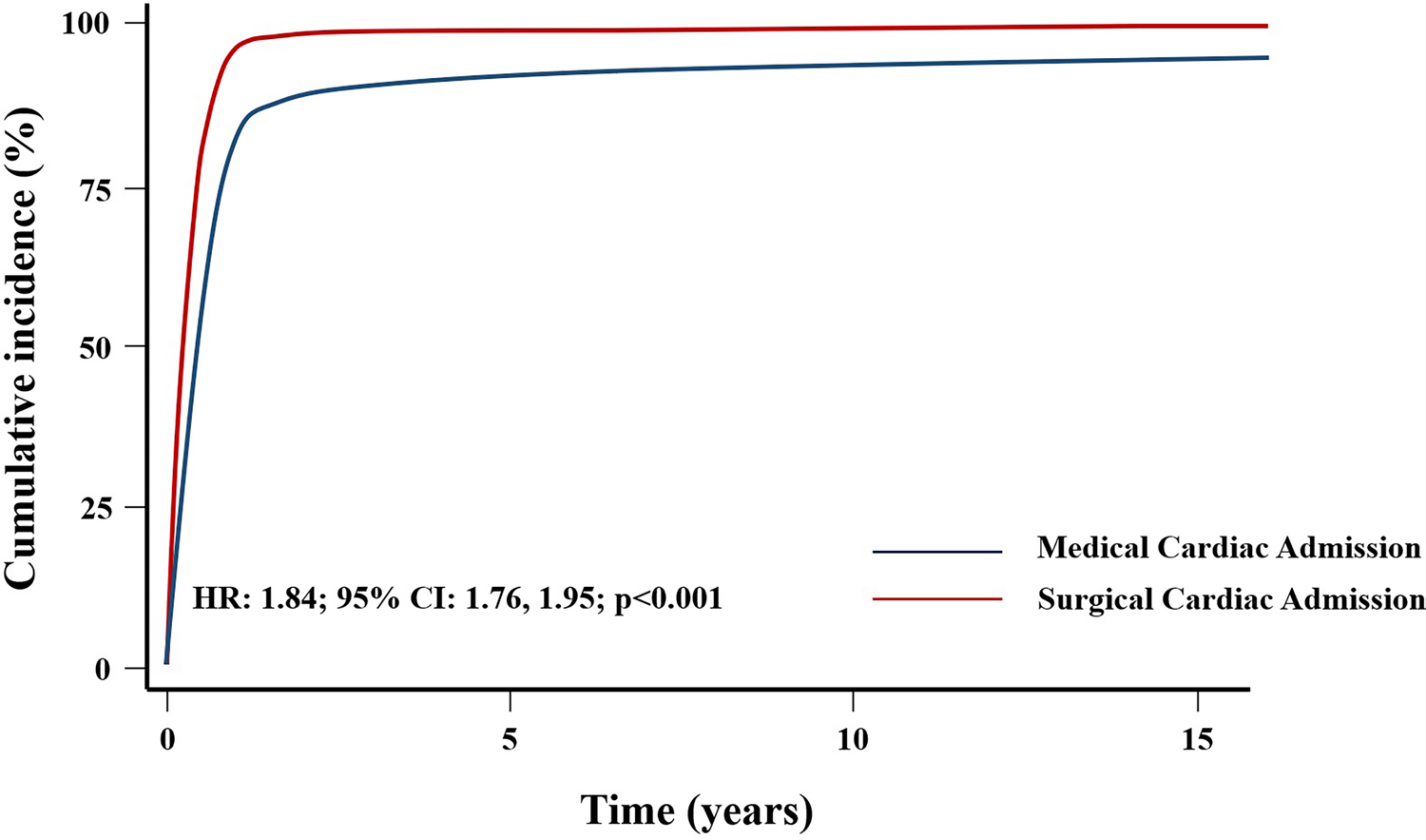
Competing Risk Modulated Renewal Regression Based on The Type of Cardiac Admission

Cox’s analysis unveiled age at first admission as the sole predictor of mortality. An increased risk of death was identified in patients with at least one cardiac admission (HR: 1.45, 95% CI 1.08–1.94, *p* = 0.012) (Fig. 3), as well as those undergoing cardiac surgery (HR = 1.51, 95% CI 1.13–2.03, *p* = 0.006) (Fig. 4), in contrast to those with non-surgical cardiac-related admissions. The study did not observe an impact of the overall number of hospital admissions on survival (HR = 1.08, 95% CI 0.81–1.44, *p* = 0.6) (Fig. 5). However, having more than one admission was associated to an increased risk of further readmissions (HR = 3.13, 95% CI 2.99, 3.27, *p* < 0.001) (Fig. 6). A similar risk of readmissions was observed for patients with at least one cardiac admission (HR = 2.00, 95% CI 1.92, 2.09, *p* < 0.001) (Fig. 7) or a history of cardiac surgery (HR = 1.84, 95% CI 1.76, 1.95, *p* < 0.001) (Fig. 8).

The study recorded in-hospital fatalities within a cohort of 216 patients, constituting 3.9% of the total, with 196 (90.7%) of these occurrences involving patients under the age of 1. The overall mortality exhibited a decline over the course of the study. However, when scrutinizing specific diagnoses, no statistically significant variances were discerned between the two temporal segments.

In the realm of various cardiac diseases, AVSD emerged as the primary contributor to mortality. This was observed both in the entire population born between 2001 and 2016 and in the subgroup subjected to cardiac surgery procedures: 117 patients, representing 54.2% of all deaths, and 59 patients, accounting for 70.2% of the deceased patients, respectively.

## Discussion

The correlation between Down syndrome and CHD constitutes a recognized risk element contributing to heightened morbidity and mortality [6, 13, 14]. Nevertheless, comprehensive nationwide statistics on hospital outcomes remain absent or scarce [7, 9, 10, 15, 16]. This study endeavors to elucidate the health ramifications for Italian individuals with Down syndrome and CHD, drawing on a dataset from HDF spanning the years 2001 to 2016.

We identified a comprehensive cohort comprising 6527 individuals affected by both Down syndrome and CHD, each of whom experienced at least one hospitalization in Italy between 2001 and 2016. Within this cohort, 4872 individuals were born during the designated study period. Our investigation validated prevailing literature findings regarding the prevalence of specific CHD subtypes within the Down syndrome population [4, 8, 9, 17, 18]. Primary among these was AVSD, followed by VSDs and ASDs. In this context, the study corroborated gender-based variations in the prevalence of certain CHDs. AVSD exhibited greater occurrence in females, while TOF predominated in males.

The distribution of surgical interventions mirrored the prevalence distribution of CHDs within the Down syndrome cohort [19]. The most frequent surgical procedure involved AVSD correction, succeeded by VSD correction, PDA closure, and ASD correction. Notably, these procedures were predominantly carried out during the pediatric phase [11, 20].

Commencing in 2012, a discernible decline in hospital admissions was observed. This trend is likely attributed to a rise in elective pregnancy terminations, especially in instances of more severe anomalies—a correlation previously substantiated [18, 21]. Intriguingly, a German study documented an upsurge in the occurrence of CHDs among individuals with DSP beginning in 2010 [18]. This prompts speculation that the heightened prevalence of pregnancies involving complex CHDs could have led to a proportional rise in cases of DSP with less intricate CHDs. Such simpler CHDs typically entail fewer hospital admissions. Nevertheless, the reduction in DSP born with a CHD during the study period did not reach statistical significance. This observation may be linked to a higher proportion of patients originating from countries outside of Italy.

Despite the wide array of concurrent health issues associated with Trisomy 21, several studies have indicated that this syndrome does not serve as a predictive factor for mortality in cardiac surgeries, except for cases involving TOF with pulmonary atresia, as well as individuals undergoing bidirectional Glenn shunt procedures [22]. Within our own study, cardiac surgery accounted for the primary reason for initial hospitalization in 9.2% of patients, with 90.1% of our study population undergoing such procedures within their first year of life. Notably, the age at which patients were first admitted to the hospital was generally lower among DSP and CHD. This trend likely reflects an effort to preempt the development of irreversible pulmonary vascular diseases, which tend to manifest earlier and more severely within this population. Additionally, this early intervention may contribute to mitigating issues related to suboptimal growth.

A significant majority, accounting for 10,701 (86.6%) of all hospital admissions were recorded during the pediatric phase, aligning with findings observed in other studies that indicate a heightened vulnerability to hospitalization during the initial years of life in individuals with Down syndrome and CHD. A notable 39.9% of pediatric patients underwent multiple hospital admissions, with an average length of stay of 12.3 ± 19.6 days. These figures are in concordance with existing literature data, which also highlight an increased likelihood of multiple hospital admissions and prolonged stays for DSP patients with CHD [16, 22–27]. This pattern may be attributed to the presence of numerous associated pathologies, which indeed account for 52.7% of all hospital admissions in our cohort. Cardiac-related admissions constituted the remaining 47.3% of cases.

This observation likely stems from the intricate range of health conditions prevalent among DSP with CHD, which collectively contribute significantly to a substantial portion of hospitalizations, extending beyond the realm of cardiac issues. Interestingly, we discerned through multivariate analysis accounting for mortality that multiple hospital admissions or the occurrence of at least one cardiac-related admission emerge as risk factors associated to diminished freedom from subsequent admissions. This data serves to underscore the pronounced burden of CHD within the clinical history of individuals with Down syndrome, reinforcing the significance of this comorbidity.

Finally, the rate of in-hospital mortality was recorded at 2.29% (*n* = 283). Through Cox analysis, it was revealed that the age at the first hospital admission emerged as a risk factor for early mortality. Moreover, diminished survival rates were evident among patients who experienced cardiac-related admissions, as well as among those who underwent at least one cardiac surgical procedure. These findings underscore the significant impact of age at first hospitalization and cardiac-related factors on the risk of mortality in our cohort.

### Limitations

This study constitutes a retrospective analysis utilizing data extracted from the Italian Ministry of Health’s HDF. It is important to acknowledge that this dataset may exhibit some heterogeneity in its compilation and does not encompass information from outpatient services. Additionally, the absence of a control cohort for comparison within the same timeframe is noteworthy. Furthermore, the information provided by the HDF lacks specific details about participating hospitals, such as their number and type, which could contribute to patient outcomes. Despite these limitations, it is essential to underscore that this investigation presents the most comprehensive examination of hospital outcomes data within the Italian Down syndrome population.

## Conclusions

Congenital heart diseases represent a significant burden in terms concerning mortality and morbidity within the population of Down syndrome patients. They contribute to an elevated frequency of hospital admissions while concurrently diminishing the freedom from further hospital admission and death. This study may provide valuable insights that could aid societies in formulating guidelines for the management and ongoing care of individuals with Down syndrome and congenital heart diseases.

## Data Availability

Data were sourced from the Hospital Discharge Form (HDF), curated by the Italian Ministry of Health and made available after 5 years. The HDF encompasses a spectrum of personal, clinical, and admission-related information, with each patient being uniquely identified through a designated code. Diagnoses and procedures outlined in the HDF were classified in accordance with the International Classification of Diseases - 9th Revision - Clinical Modification System (ICD-9-CM).
